# Supplementary material for: The Effect of Limosilactobacillus fermentum MG4717 on Oral Health and Biosafety
Source: Microorganisms. 2025 Jul 7;13(7):1600. doi: 10.3390/microorganisms13071600 (PMC12299399; doi:10.3390/microorganisms13071600)
Supplement: Supplementary file 1 [file microorganisms-13-01600-s001.zip › microorganisms-3707010-supplementary.pdf]

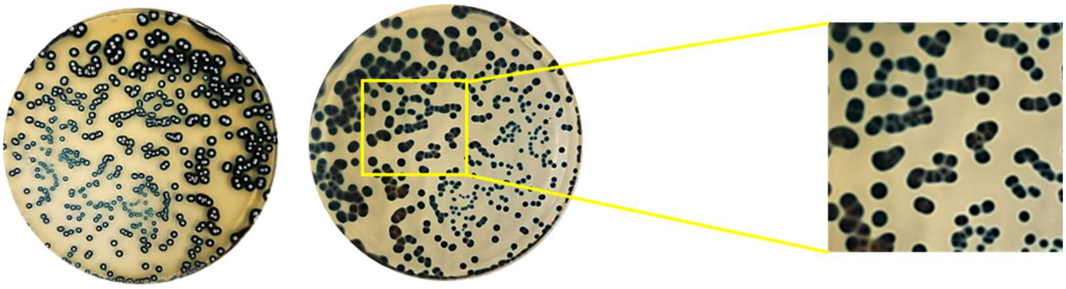

Figure S1. $H_2O2_2$ production of *L. fermentum* MG4717. The blue colonies on the TMB agar would be categorized as $H_2O_2$-positive.
